# Bis[bis­(1-ethyl­benzimidazol-2-ylmeth­yl) ether]cobalt(II) dipicrate dimethyl­formamide disolvate

**DOI:** 10.1107/S1600536809035934

**Published:** 2009-09-12

**Authors:** Huilu Wu, Ruirui Yun, Tao Sun, Ke Li, Xuan Meng

**Affiliations:** a School of Chemical and Biological Engineering, Lanzhou Jiaotong University, Lanzhou 730070, People’s Republic of China

## Abstract

In the title complex, [Co(C_20_H_22_N_4_O)_2_](C_6_H_2_N_3_O_7_)_2_·2C_3_H_7_NO, the Co^II^ ion is coordinated by two sets of two N atoms and an O atom from two independent tridendate ligands in a distorted octa­hedral coordination environment. There are significant differences between chemically equivalent coordination bond lengths. The crystal structure is stabilized by weak inter­molecular C—H⋯O hydrogen bonds and weak π–π stacking inter­actions [centroid–centroid distance 3.495 (1) Å]. In one of the anions one nitro group is rotationally disordered about the C—N bond with refined occupancies of 0.524 (8) and 0.476 (8).

## Related literature

For related structures, see: Wu, Yun, Huang *et al.* (2009 ▶); Wu, Yun, Li *et al.* (2009 ▶); Yun *et al.* (2008 ▶). 
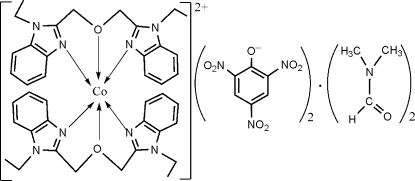


## Experimental

### 

#### Crystal data

[Co(C_20_H_22_N_4_O)_2_](C_6_H_2_N_3_O_7_)_2_·2C_3_H_7_NO
*M* *_r_* = 1330.17
Triclinic, 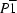

*a* = 13.8799 (4) Å
*b* = 14.6525 (4) Å
*c* = 16.5701 (3) Å
α = 110.664 (1)°
β = 96.470 (1)°
γ = 99.612 (1)°
*V* = 3054.62 (13) Å^3^
*Z* = 2
Mo *K*α radiation
μ = 0.37 mm^−1^
*T* = 153 K
0.38 × 0.22 × 0.14 mm

#### Data collection

Rigaku R-AXIS Spider diffractometer
Absorption correction: multi-scan (*ABSCOR*; Higashi, 1995 ▶) *T*_min_ = 0.873, *T*_max_ = 0.950
24337 measured reflections
11225 independent reflections
8833 reflections with *I* > 2σ(*I*)
*R*_int_ = 0.022

#### Refinement

*R*[*F* ^2^ > 2σ(*F* ^2^)] = 0.042
*wR*(*F* ^2^) = 0.136
*S* = 1.14
11225 reflections
858 parameters
4 restraints
H-atom parameters constrained
Δρ_max_ = 0.98 e Å^−3^
Δρ_min_ = −0.90 e Å^−3^

### 

Data collection: *RAPID-AUTO* (Rigaku/MSC 2004 ▶); cell refinement: *RAPID-AUTO*; data reduction: *RAPID-AUTO*; program(s) used to solve structure: *SHELXS97* (Sheldrick, 2008 ▶); program(s) used to refine structure: *SHELXL97* (Sheldrick, 2008 ▶); molecular graphics: *SHELXTL* (Sheldrick, 2008 ▶) and *PLATON* (Spek, 2009 ▶); software used to prepare material for publication: *SHELXTL*.

## Supplementary Material

Crystal structure: contains datablocks global, I. DOI: 10.1107/S1600536809035934/lh2889sup1.cif

Structure factors: contains datablocks I. DOI: 10.1107/S1600536809035934/lh2889Isup2.hkl

Additional supplementary materials:  crystallographic information; 3D view; checkCIF report

## Figures and Tables

**Table:** 

Co—N1	2.083 (2)
Co—N3	2.099 (2)
Co—N7	2.108 (2)
Co—N5	2.174 (2)
Co—O2	2.1961 (17)
Co—O1	2.2872 (16)

**Table:** 

N1—Co—N3	140.20 (8)
N1—Co—N7	99.60 (8)
N3—Co—N7	100.49 (8)
N1—Co—N5	97.14 (8)
N3—Co—N5	85.56 (8)
N7—Co—N5	144.80 (7)
N1—Co—O2	103.97 (8)
N3—Co—O2	114.58 (7)
N7—Co—O2	73.35 (7)
N5—Co—O2	72.66 (7)
N1—Co—O1	72.54 (7)
N3—Co—O1	71.85 (7)
N7—Co—O1	94.77 (7)
N5—Co—O1	119.81 (7)
O2—Co—O1	167.10 (7)

**Table 2:** Hydrogen-bond geometry (Å, °)

*D*—H⋯*A*	*D*—H	H⋯*A*	*D*⋯*A*	*D*—H⋯*A*
C18—H18*B*⋯O12^i^	0.98	2.57	3.342 (4)	135
C15—H15*A*⋯O13	0.95	2.56	3.191 (3)	124
C18—H18*B*⋯O12^i^	0.98	2.57	3.342 (4)	135
C28—H28*A*⋯O17^ii^	0.99	2.38	3.365 (4)	173
C28—H28*B*⋯O10^iii^	0.99	2.29	2.994 (3)	128
C29—H29*A*⋯O14	0.99	2.39	3.331 (3)	158
C35—H35*A*⋯O7^i^	0.95	2.44	3.158 (3)	133
C53—H53⋯O9′^iv^	0.95	2.39	3.242 (7)	148
C56—H56*A*⋯O3^v^	0.95	2.46	3.335 (4)	154

Symmetry codes: (i) 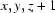; (ii) 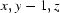; (iii) 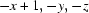; (iv) 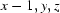; (v) 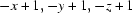.

**Fig. 1.**
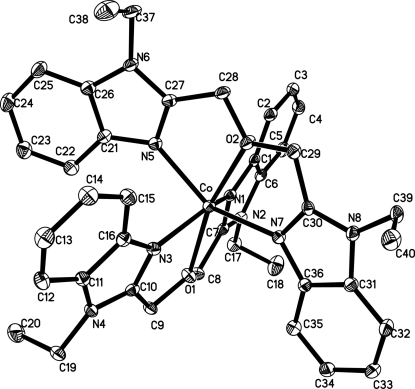
Molecular structure and atom numbering for the cation of the title compound. Hydrogen atoms have been omitted for clarity and the displacement ellipsoids are shown at the 30% probability level.

**Fig. 2.**
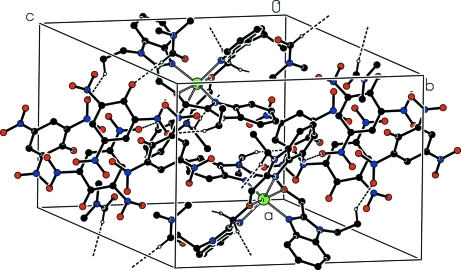
Part of the crystal structure showing weak C—H···O hydrogen bonds (dashed lines) and weak π···π stacking interactions. Only H atoms involved in hydrogen bonds are shown. The disorder is not shown.

## supplementary crystallographic information

### Comment

Interest in bis(2-benzimidazolyl)alkanes and their derivatives are widespread and we have previously reported the crystal structure of some related complexes (Wu, Yun, Huang *et al.*, 2009; Wu, Yun, Li *et al.*, 2009; Yun *et al.*, 2008). The asymmetric unit of the title compound consists of a di[1,3-bis(1-ethylbenzimidazol-2-yl)-2-oxopropane] cobalt(II) cation (Fig. 1), two picrate anions and two molecules of DMF. The Co^II^ ion is six-coordinated with a N_4_O_2_ ligand set. The Etobb (1,3-bis(1-ethylbenzimidazol-2-yl)-2-oxopropane) ligand acts as a tridentate donor. The four N atoms are not in an ideal equatorial plane and the coordination geometry of the Co^II^ may be best described as distorted octahedral. There are significant differnces between chemically equivalent coordination bond lengths. The crystal structure is stabilized by weak intermolecular C—H···O hydrogen bond interactions and weak π···π stacking interactions (Fig. 2) with centroid to centroid distances of 3.495 (1)Å  between inversion related benzimidazole rings systems.

### Experimental

To a stirred solution of 1,3-bis(1-ethylbenzimidazol-2-yl)-2-oxopropane (0.167 g, 0.5 mmol) in hot MeOH (15 ml) was added Co(C_6_H_2_N_3_O_7_)_2_ (0.129 g, 0.25 mmol) in MeOH (5 ml). A brown crystalline product formed rapidly. The precipitate was filtered off, washed with MeOH and absolute Et_2_O, and dried *in vacuo*. The dried precipitate was dissolved in DMF resulting in a brown solution. Brown crystals suitable for X-ray diffraction studies were obtained after three days at room temperature from ether diffusion into a DMF solution of the title compound. Yield, 0.210 g (71%). (found: C, 52.41; H, 4.68; N, 16.81. Calcd. for C116H124Co2N32O36: C, 52.37; H, 4.70; N, 16.85)

### Refinement

All H atoms were found in difference electron maps and were subsequently refined in a riding-model approximation with C—H distances ranging from 0.95 to 0.99 Å and *U*_iso_(H) = 1.2 *U*_eq_ or *U*_iso_(H) = 1.5*U*_eq_ of the carrier atom, respectively. In one of the anions one nitro group is rotationally disordered about the C-N bond with refined occupancies of 0.524 (8) [O8,O9] and 0.476 (8) [O8',O9'].

### Crystal data

**Table:**

[Co(C_20_H_22_N_4_O)_2_](C_6_H_2_N_3_O_7_)_2_·2C_3_H_7_NO	*Z* = 2
*M**_r_* = 1330.17	*F*(000) = 1386
Triclinic, *P*1	*D*_x_ = 1.446 Mg m^−^^3^
Hall symbol: -P 1	Mo *K*α radiation, λ = 0.71073 Å
*a* = 13.8799 (4) Å	Cell parameters from 7749 reflections
*b* = 14.6525 (4) Å	θ = 3.0–25.5°
*c* = 16.5701 (3) Å	µ = 0.37 mm^−^^1^
α = 110.664 (1)°	*T* = 153 K
β = 96.470 (1)°	Block, brown
γ = 99.612 (1)°	0.38 × 0.22 × 0.14 mm
*V* = 3054.62 (13) Å^3^	

### Data collection

**Table:**

Rigaku R-AXIS Spider diffractometer	11225 independent reflections
Radiation source: fine-focus sealed tube	8833 reflections with *I* > 2σ(*I*)
graphite	*R*_int_ = 0.022
φ and ω scans	θ_max_ = 25.5°, θ_min_ = 3.0°
Absorption correction: multi-scan (*ABSCOR*; Higashi, 1995)	*h* = −16→16
*T*_min_ = 0.873, *T*_max_ = 0.950	*k* = −17→17
24337 measured reflections	*l* = −18→20

### Refinement

**Table:**

Refinement on *F*^2^	Secondary atom site location: difference Fourier map
Least-squares matrix: full	Hydrogen site location: inferred from neighbouring sites
*R*[*F*^2^ > 2σ(*F*^2^)] = 0.042	H-atom parameters constrained
*wR*(*F*^2^) = 0.136	*w* = 1/[σ^2^(*F*_o_^2^) + (0.0663*P*)^2^ + 2.0559*P*] where *P* = (*F*_o_^2^ + 2*F*_c_^2^)/3
*S* = 1.14	(Δ/σ)_max_ = 0.10
11225 reflections	Δρ_max_ = 0.98 e Å^−^^3^
858 parameters	Δρ_min_ = −0.89 e Å^−^^3^
4 restraints	Extinction correction: *SHELXL*, Fc^*^=kFc[1+0.001xFc^2^λ^3^/sin(2θ)]^-1/4^
Primary atom site location: structure-invariant direct methods	Extinction coefficient: 0.0022 (5)

### Special details

**Table:**

Geometry. All e.s.d.'s (except the e.s.d. in the dihedral angle between two l.s. planes) are estimated using the full covariance matrix. The cell e.s.d.'s are taken into account individually in the estimation of e.s.d.'s in distances, angles and torsion angles; correlations between e.s.d.'s in cell parameters are only used when they are defined by crystal symmetry. An approximate (isotropic) treatment of cell e.s.d.'s is used for estimating e.s.d.'s involving l.s. planes.
Refinement. Refinement of *F*^2^ against ALL reflections. The weighted *R*-factor *wR* and goodness of fit *S* are based on *F*^2^, conventional *R*-factors *R* are based on *F*, with *F* set to zero for negative *F*^2^. The threshold expression of *F*^2^ > σ(*F*^2^) is used only for calculating *R*-factors(gt) *etc*. and is not relevant to the choice of reflections for refinement. *R*-factors based on *F*^2^ are statistically about twice as large as those based on *F*, and *R*- factors based on ALL data will be even larger.

### Fractional atomic coordinates and isotropic or equivalent isotropic displacement parameters (Å^2^)

**Table:**

	*x*	*y*	*z*	*U*_iso_*/*U*_eq_	Occ. (<1)
Co	0.28649 (2)	0.19838 (2)	0.46816 (2)	0.01823 (11)	
O1	0.29902 (13)	0.30499 (13)	0.61018 (11)	0.0234 (4)	
O2	0.29752 (13)	0.08087 (14)	0.34517 (11)	0.0278 (4)	
O3	0.80117 (19)	0.5715 (2)	0.13764 (15)	0.0629 (7)	
O4	0.5964 (3)	0.3781 (2)	0.17574 (16)	0.0752 (10)	
O5	0.7548 (3)	0.4037 (3)	0.1790 (2)	0.0980 (12)	
O6	0.41385 (18)	0.2944 (2)	−0.11930 (18)	0.0619 (7)	
O7	0.45343 (19)	0.3970 (2)	−0.18404 (14)	0.0559 (7)	
O8	0.7909 (5)	0.6140 (5)	−0.0805 (3)	0.082 (3)	0.524 (8)
O9	0.8201 (4)	0.6944 (5)	0.0537 (4)	0.064 (2)	0.524 (8)
O8'	0.7091 (5)	0.6837 (5)	−0.0389 (4)	0.089 (3)	0.476 (8)
O9'	0.8490 (4)	0.6593 (10)	0.0175 (9)	0.116 (4)	0.476 (8)
O10	0.67849 (16)	0.05378 (17)	−0.11405 (13)	0.0406 (5)	
O11	0.54090 (17)	0.13588 (18)	−0.18370 (13)	0.0434 (5)	
O12	0.40246 (16)	0.06268 (18)	−0.16289 (14)	0.0443 (5)	
O13	0.39642 (17)	0.17501 (17)	0.15142 (14)	0.0442 (5)	
O14	0.52461 (17)	0.17492 (16)	0.23989 (12)	0.0407 (5)	
O15	0.80210 (18)	0.0547 (2)	0.11187 (18)	0.0587 (7)	
O16	0.84422 (16)	0.1175 (2)	0.01821 (15)	0.0518 (6)	
O17	0.10881 (18)	0.8163 (2)	0.20909 (15)	0.0575 (7)	
O18	0.07622 (17)	0.40600 (18)	0.63580 (14)	0.0473 (6)	
N1	0.20049 (14)	0.12155 (16)	0.52909 (12)	0.0197 (4)	
N2	0.14353 (15)	0.10419 (16)	0.64503 (13)	0.0223 (5)	
N3	0.33896 (15)	0.34492 (15)	0.47641 (13)	0.0200 (4)	
N4	0.36761 (15)	0.51053 (15)	0.54120 (13)	0.0209 (4)	
N5	0.16690 (15)	0.19040 (16)	0.36748 (13)	0.0206 (4)	
N6	0.09391 (16)	0.13339 (17)	0.22634 (13)	0.0248 (5)	
N7	0.42207 (15)	0.15693 (15)	0.49147 (12)	0.0196 (4)	
N8	0.54212 (16)	0.07996 (16)	0.44281 (13)	0.0235 (5)	
N9	0.6710 (3)	0.4043 (2)	0.14874 (18)	0.0558 (9)	
N10	0.4692 (2)	0.3651 (2)	−0.12503 (18)	0.0426 (7)	
N11	0.7593 (3)	0.6329 (3)	−0.01009 (19)	0.0867 (15)	
N12	0.49148 (18)	0.10217 (18)	−0.13969 (14)	0.0321 (5)	
N13	0.48254 (19)	0.16547 (18)	0.16615 (15)	0.0327 (6)	
N14	0.78255 (18)	0.0908 (2)	0.05747 (16)	0.0370 (6)	
N15	0.1137 (2)	0.7045 (3)	0.07460 (19)	0.0623 (9)	
N16	−0.0601 (2)	0.4302 (2)	0.69697 (17)	0.0453 (7)	
C1	0.14755 (17)	0.02311 (19)	0.50421 (16)	0.0202 (5)	
C2	0.12581 (18)	−0.0567 (2)	0.42273 (16)	0.0237 (5)	
H2A	0.1477	−0.0491	0.3727	0.028*	
C3	0.07159 (19)	−0.1465 (2)	0.41792 (18)	0.0278 (6)	
H3A	0.0565	−0.2020	0.3635	0.033*	
C4	0.0378 (2)	−0.1586 (2)	0.49117 (18)	0.0289 (6)	
H4A	0.0010	−0.2222	0.4853	0.035*	
C5	0.05712 (19)	−0.0795 (2)	0.57183 (18)	0.0282 (6)	
H5A	0.0339	−0.0869	0.6214	0.034*	
C6	0.11200 (18)	0.01088 (19)	0.57638 (16)	0.0220 (5)	
C7	0.19536 (17)	0.16562 (19)	0.61295 (15)	0.0194 (5)	
C8	0.24117 (19)	0.27325 (19)	0.66549 (15)	0.0224 (5)	
H8A	0.1893	0.3122	0.6799	0.027*	
H8B	0.2838	0.2815	0.7208	0.027*	
C9	0.3238 (2)	0.40958 (18)	0.63268 (15)	0.0229 (5)	
H9A	0.3830	0.4412	0.6802	0.028*	
H9B	0.2677	0.4400	0.6523	0.028*	
C10	0.34504 (17)	0.42222 (19)	0.55015 (15)	0.0206 (5)	
C11	0.37509 (18)	0.48952 (19)	0.45373 (16)	0.0223 (5)	
C12	0.3948 (2)	0.5512 (2)	0.40767 (18)	0.0291 (6)	
H12A	0.4070	0.6220	0.4351	0.035*	
C13	0.3958 (2)	0.5032 (2)	0.31901 (18)	0.0346 (7)	
H13A	0.4087	0.5425	0.2848	0.041*	
C14	0.3782 (2)	0.3990 (2)	0.27845 (18)	0.0315 (6)	
H14A	0.3796	0.3693	0.2177	0.038*	
C15	0.3590 (2)	0.3387 (2)	0.32506 (17)	0.0271 (6)	
H15A	0.3475	0.2679	0.2977	0.033*	
C16	0.35722 (18)	0.38544 (19)	0.41382 (16)	0.0221 (5)	
C17	0.1169 (2)	0.1340 (2)	0.73351 (17)	0.0282 (6)	
H17A	0.0446	0.1088	0.7274	0.034*	
H17B	0.1312	0.2079	0.7612	0.034*	
C18	0.1725 (3)	0.0947 (3)	0.7923 (2)	0.0495 (9)	
H18A	0.1522	0.1163	0.8497	0.074*	
H18B	0.2441	0.1207	0.7998	0.074*	
H18C	0.1576	0.0215	0.7659	0.074*	
C19	0.3731 (2)	0.60909 (19)	0.60915 (16)	0.0263 (6)	
H19A	0.4201	0.6604	0.5984	0.032*	
H19B	0.3991	0.6091	0.6673	0.032*	
C20	0.2727 (2)	0.6357 (2)	0.6101 (2)	0.0423 (8)	
H20A	0.2791	0.7016	0.6563	0.063*	
H20B	0.2262	0.5855	0.6215	0.063*	
H20C	0.2475	0.6374	0.5531	0.063*	
C21	0.09872 (18)	0.24717 (19)	0.35843 (15)	0.0217 (5)	
C22	0.0729 (2)	0.3280 (2)	0.42065 (17)	0.0274 (6)	
H22A	0.1039	0.3535	0.4807	0.033*	
C23	0.0009 (2)	0.3692 (2)	0.39152 (19)	0.0341 (7)	
H23A	−0.0183	0.4237	0.4326	0.041*	
C24	−0.0450 (2)	0.3330 (2)	0.30304 (19)	0.0363 (7)	
H24A	−0.0944	0.3636	0.2857	0.044*	
C25	−0.0199 (2)	0.2536 (2)	0.24042 (19)	0.0332 (6)	
H25A	−0.0503	0.2289	0.1802	0.040*	
C26	0.05232 (19)	0.2121 (2)	0.27061 (16)	0.0251 (6)	
C27	0.16210 (18)	0.12508 (19)	0.28700 (15)	0.0221 (5)	
C28	0.22980 (19)	0.0551 (2)	0.26444 (16)	0.0259 (6)	
H28A	0.1923	−0.0153	0.2432	0.031*	
H28B	0.2658	0.0642	0.2186	0.031*	
C29	0.38727 (18)	0.0477 (2)	0.33422 (16)	0.0233 (5)	
H29A	0.4191	0.0695	0.2916	0.028*	
H29B	0.3748	−0.0261	0.3134	0.028*	
C30	0.45091 (18)	0.09541 (18)	0.42356 (15)	0.0195 (5)	
C31	0.57725 (19)	0.13738 (19)	0.53148 (16)	0.0233 (5)	
C32	0.6663 (2)	0.1502 (2)	0.58537 (18)	0.0298 (6)	
H32A	0.7174	0.1183	0.5633	0.036*	
C33	0.6775 (2)	0.2111 (2)	0.67244 (18)	0.0306 (6)	
H33A	0.7376	0.2219	0.7115	0.037*	
C34	0.6014 (2)	0.2577 (2)	0.70433 (17)	0.0286 (6)	
H34A	0.6113	0.2992	0.7647	0.034*	
C35	0.51260 (19)	0.2449 (2)	0.65041 (16)	0.0251 (6)	
H35A	0.4612	0.2760	0.6727	0.030*	
C36	0.50137 (18)	0.18448 (19)	0.56205 (15)	0.0204 (5)	
C37	0.0750 (2)	0.0776 (2)	0.13075 (16)	0.0333 (7)	
H37A	0.0028	0.0625	0.1076	0.040*	
H37B	0.0957	0.0133	0.1182	0.040*	
C38	0.1305 (3)	0.1359 (3)	0.0843 (2)	0.0502 (9)	
H38A	0.1163	0.0963	0.0211	0.075*	
H38B	0.2021	0.1501	0.1065	0.075*	
H38C	0.1090	0.1989	0.0954	0.075*	
C39	0.6008 (2)	0.0221 (2)	0.38379 (17)	0.0279 (6)	
H39A	0.5560	−0.0255	0.3289	0.033*	
H39B	0.6329	−0.0173	0.4121	0.033*	
C40	0.6796 (2)	0.0891 (2)	0.36196 (19)	0.0363 (7)	
H40A	0.7171	0.0482	0.3226	0.055*	
H40B	0.7249	0.1353	0.4161	0.055*	
H40C	0.6479	0.1273	0.3330	0.055*	
C41	0.7297 (2)	0.5228 (2)	0.07784 (18)	0.0378 (7)	
C42	0.6578 (2)	0.4375 (2)	0.07595 (17)	0.0350 (7)	
C43	0.5747 (2)	0.3879 (2)	0.01257 (18)	0.0330 (6)	
H43A	0.5291	0.3348	0.0173	0.040*	
C44	0.5576 (2)	0.4155 (2)	−0.05849 (17)	0.0310 (6)	
C45	0.6213 (2)	0.4953 (2)	−0.06346 (17)	0.0367 (7)	
H45A	0.6087	0.5148	−0.1118	0.044*	
C46	0.7027 (2)	0.5462 (2)	0.00134 (19)	0.0388 (7)	
C47	0.6395 (2)	0.0877 (2)	−0.04959 (17)	0.0278 (6)	
C48	0.5411 (2)	0.1095 (2)	−0.05470 (16)	0.0265 (6)	
C49	0.4898 (2)	0.1320 (2)	0.01257 (16)	0.0264 (6)	
H49A	0.4240	0.1413	0.0034	0.032*	
C50	0.5358 (2)	0.1411 (2)	0.09506 (16)	0.0261 (6)	
C51	0.6320 (2)	0.1276 (2)	0.10885 (17)	0.0293 (6)	
H51A	0.6629	0.1342	0.1654	0.035*	
C52	0.6820 (2)	0.1045 (2)	0.04027 (17)	0.0286 (6)	
C53	0.0698 (3)	0.7568 (4)	0.1365 (3)	0.0910 (19)	
H53	−0.0004	0.7463	0.1222	0.109*	
C54	0.2175 (3)	0.7168 (3)	0.0886 (2)	0.0534 (9)	
H54A	0.2460	0.7619	0.1493	0.080*	
H54B	0.2447	0.7454	0.0484	0.080*	
H54C	0.2344	0.6517	0.0777	0.080*	
C55	0.0620 (4)	0.6385 (6)	−0.0135 (4)	0.162 (4)	
H55A	−0.0096	0.6347	−0.0175	0.242*	
H55B	0.0749	0.5715	−0.0262	0.242*	
H55C	0.0860	0.6648	−0.0561	0.242*	
C56	0.0356 (2)	0.4296 (2)	0.6990 (2)	0.0416 (8)	
H56A	0.0762	0.4495	0.7551	0.050*	
C57	−0.1274 (3)	0.4030 (3)	0.6141 (2)	0.0497 (8)	
H57A	−0.0914	0.3820	0.5656	0.075*	
H57B	−0.1538	0.4608	0.6136	0.075*	
H57C	−0.1825	0.3478	0.6074	0.075*	
C58	−0.1011 (4)	0.4648 (4)	0.7763 (3)	0.099 (2)	
H58A	−0.0493	0.4805	0.8275	0.149*	
H58B	−0.1562	0.4123	0.7746	0.149*	
H58C	−0.1255	0.5250	0.7803	0.149*	

### Atomic displacement parameters (Å^2^)

**Table:**

	*U*^11^	*U*^22^	*U*^33^	*U*^12^	*U*^13^	*U*^23^
Co	0.01811 (18)	0.0209 (2)	0.01589 (17)	0.00583 (13)	0.00398 (12)	0.00627 (13)
O1	0.0314 (10)	0.0197 (9)	0.0205 (9)	0.0050 (8)	0.0116 (7)	0.0075 (7)
O2	0.0226 (10)	0.0405 (12)	0.0179 (9)	0.0166 (8)	0.0030 (7)	0.0040 (8)
O3	0.0526 (16)	0.0693 (18)	0.0400 (13)	−0.0042 (13)	−0.0136 (11)	0.0035 (12)
O4	0.139 (3)	0.0478 (16)	0.0359 (14)	0.0037 (17)	0.0277 (16)	0.0170 (12)
O5	0.126 (3)	0.106 (3)	0.068 (2)	0.041 (2)	−0.024 (2)	0.0462 (19)
O6	0.0374 (14)	0.0474 (16)	0.0740 (18)	0.0025 (12)	−0.0019 (12)	−0.0022 (13)
O7	0.0561 (16)	0.0713 (18)	0.0288 (12)	0.0351 (14)	−0.0065 (10)	0.0000 (11)
O8	0.089 (5)	0.081 (5)	0.070 (4)	−0.009 (4)	0.038 (3)	0.028 (3)
O9	0.033 (3)	0.051 (4)	0.093 (5)	−0.008 (3)	0.000 (3)	0.020 (3)
O8'	0.115 (6)	0.078 (5)	0.076 (5)	−0.031 (4)	−0.014 (4)	0.065 (4)
O9'	0.072 (6)	0.120 (10)	0.160 (11)	−0.004 (6)	0.033 (6)	0.066 (8)
O10	0.0462 (13)	0.0484 (14)	0.0297 (11)	0.0229 (11)	0.0162 (9)	0.0094 (9)
O11	0.0488 (13)	0.0599 (15)	0.0291 (11)	0.0127 (11)	0.0105 (9)	0.0248 (10)
O12	0.0330 (13)	0.0553 (15)	0.0398 (12)	0.0022 (11)	−0.0048 (9)	0.0194 (10)
O13	0.0399 (13)	0.0526 (15)	0.0425 (12)	0.0184 (11)	0.0209 (10)	0.0132 (10)
O14	0.0554 (14)	0.0444 (13)	0.0219 (10)	0.0091 (11)	0.0129 (9)	0.0113 (9)
O15	0.0397 (14)	0.085 (2)	0.0775 (18)	0.0222 (13)	0.0072 (12)	0.0592 (16)
O16	0.0307 (12)	0.0788 (19)	0.0536 (14)	0.0140 (12)	0.0156 (10)	0.0312 (13)
O17	0.0457 (14)	0.0662 (17)	0.0402 (13)	0.0142 (13)	0.0072 (11)	−0.0046 (12)
O18	0.0415 (13)	0.0523 (15)	0.0379 (12)	0.0189 (11)	0.0043 (10)	0.0022 (10)
N1	0.0158 (10)	0.0250 (12)	0.0176 (10)	0.0064 (9)	0.0028 (8)	0.0065 (8)
N2	0.0191 (11)	0.0300 (12)	0.0197 (10)	0.0054 (9)	0.0062 (8)	0.0110 (9)
N3	0.0202 (11)	0.0198 (11)	0.0194 (10)	0.0015 (9)	0.0039 (8)	0.0079 (8)
N4	0.0234 (11)	0.0159 (11)	0.0222 (11)	0.0032 (9)	0.0038 (8)	0.0063 (8)
N5	0.0191 (11)	0.0229 (11)	0.0190 (10)	0.0051 (9)	0.0044 (8)	0.0065 (8)
N6	0.0232 (11)	0.0322 (13)	0.0166 (10)	0.0101 (10)	0.0012 (8)	0.0056 (9)
N7	0.0190 (11)	0.0223 (11)	0.0180 (10)	0.0060 (9)	0.0046 (8)	0.0072 (8)
N8	0.0220 (11)	0.0263 (12)	0.0229 (11)	0.0103 (9)	0.0046 (8)	0.0078 (9)
N9	0.099 (3)	0.0392 (17)	0.0260 (14)	0.0201 (17)	0.0034 (16)	0.0089 (12)
N10	0.0321 (15)	0.0375 (16)	0.0414 (16)	0.0165 (13)	0.0017 (11)	−0.0074 (12)
N11	0.117 (4)	0.079 (3)	0.0377 (19)	−0.042 (3)	0.007 (2)	0.0219 (18)
N12	0.0365 (14)	0.0349 (14)	0.0244 (12)	0.0123 (11)	0.0051 (10)	0.0090 (10)
N13	0.0414 (15)	0.0270 (13)	0.0284 (13)	0.0057 (11)	0.0148 (11)	0.0073 (10)
N14	0.0309 (14)	0.0438 (16)	0.0405 (14)	0.0118 (12)	0.0058 (11)	0.0196 (12)
N15	0.0422 (17)	0.064 (2)	0.0459 (17)	0.0149 (15)	−0.0057 (13)	−0.0179 (15)
N16	0.0410 (16)	0.0472 (17)	0.0354 (14)	0.0045 (13)	0.0070 (11)	0.0037 (12)
C1	0.0131 (12)	0.0231 (13)	0.0263 (13)	0.0063 (10)	0.0029 (9)	0.0110 (10)
C2	0.0159 (12)	0.0313 (15)	0.0234 (13)	0.0079 (11)	0.0034 (9)	0.0087 (11)
C3	0.0222 (14)	0.0249 (14)	0.0318 (14)	0.0080 (11)	−0.0003 (10)	0.0058 (11)
C4	0.0234 (14)	0.0220 (14)	0.0408 (16)	0.0025 (11)	0.0024 (11)	0.0138 (12)
C5	0.0218 (14)	0.0323 (16)	0.0366 (15)	0.0064 (12)	0.0060 (11)	0.0201 (12)
C6	0.0149 (12)	0.0261 (14)	0.0252 (13)	0.0055 (10)	0.0025 (9)	0.0101 (10)
C7	0.0170 (12)	0.0238 (13)	0.0188 (12)	0.0070 (10)	0.0038 (9)	0.0087 (10)
C8	0.0245 (13)	0.0247 (14)	0.0165 (12)	0.0032 (11)	0.0068 (9)	0.0062 (10)
C9	0.0290 (14)	0.0168 (13)	0.0199 (12)	0.0037 (11)	0.0040 (10)	0.0039 (10)
C10	0.0159 (12)	0.0248 (14)	0.0191 (12)	0.0043 (10)	0.0015 (9)	0.0064 (10)
C11	0.0185 (13)	0.0221 (13)	0.0259 (13)	0.0027 (10)	0.0049 (10)	0.0091 (10)
C12	0.0330 (15)	0.0230 (14)	0.0337 (15)	0.0056 (12)	0.0082 (11)	0.0133 (11)
C13	0.0389 (17)	0.0405 (18)	0.0329 (15)	0.0069 (14)	0.0124 (12)	0.0232 (13)
C14	0.0387 (16)	0.0347 (16)	0.0241 (13)	0.0079 (13)	0.0123 (11)	0.0129 (12)
C15	0.0302 (15)	0.0257 (14)	0.0254 (13)	0.0073 (12)	0.0091 (11)	0.0078 (11)
C16	0.0176 (12)	0.0256 (14)	0.0242 (13)	0.0049 (10)	0.0051 (9)	0.0105 (10)
C17	0.0264 (14)	0.0394 (17)	0.0244 (13)	0.0104 (12)	0.0111 (11)	0.0155 (12)
C18	0.059 (2)	0.073 (3)	0.0339 (17)	0.0341 (19)	0.0170 (15)	0.0302 (17)
C19	0.0324 (15)	0.0192 (14)	0.0239 (13)	0.0041 (11)	0.0059 (11)	0.0048 (10)
C20	0.0413 (18)	0.0342 (18)	0.0475 (18)	0.0171 (15)	0.0096 (14)	0.0067 (14)
C21	0.0180 (12)	0.0268 (14)	0.0215 (12)	0.0044 (11)	0.0053 (9)	0.0104 (10)
C22	0.0272 (14)	0.0306 (15)	0.0255 (13)	0.0092 (12)	0.0083 (10)	0.0096 (11)
C23	0.0337 (16)	0.0365 (17)	0.0357 (16)	0.0163 (13)	0.0127 (12)	0.0124 (13)
C24	0.0321 (16)	0.0439 (19)	0.0404 (16)	0.0198 (14)	0.0075 (12)	0.0196 (14)
C25	0.0275 (15)	0.0430 (18)	0.0307 (15)	0.0145 (13)	0.0017 (11)	0.0140 (13)
C26	0.0232 (14)	0.0293 (15)	0.0224 (13)	0.0070 (11)	0.0046 (10)	0.0085 (11)
C27	0.0183 (12)	0.0264 (14)	0.0183 (12)	0.0038 (10)	0.0024 (9)	0.0055 (10)
C28	0.0236 (14)	0.0328 (15)	0.0163 (12)	0.0096 (11)	−0.0005 (10)	0.0033 (10)
C29	0.0220 (13)	0.0281 (14)	0.0211 (12)	0.0108 (11)	0.0077 (10)	0.0076 (10)
C30	0.0198 (13)	0.0173 (13)	0.0239 (12)	0.0048 (10)	0.0066 (10)	0.0099 (10)
C31	0.0223 (13)	0.0234 (14)	0.0242 (13)	0.0047 (11)	0.0032 (10)	0.0095 (10)
C32	0.0245 (14)	0.0348 (16)	0.0336 (15)	0.0129 (12)	0.0049 (11)	0.0145 (12)
C33	0.0266 (15)	0.0321 (16)	0.0307 (14)	0.0058 (12)	−0.0032 (11)	0.0117 (12)
C34	0.0317 (15)	0.0285 (15)	0.0234 (13)	0.0062 (12)	0.0012 (11)	0.0085 (11)
C35	0.0249 (14)	0.0286 (15)	0.0239 (13)	0.0071 (11)	0.0067 (10)	0.0113 (11)
C36	0.0202 (13)	0.0205 (13)	0.0215 (12)	0.0032 (10)	0.0031 (9)	0.0100 (10)
C37	0.0302 (15)	0.0452 (18)	0.0164 (13)	0.0120 (13)	−0.0031 (10)	0.0028 (12)
C38	0.053 (2)	0.070 (3)	0.0279 (16)	0.0150 (18)	0.0104 (14)	0.0173 (16)
C39	0.0272 (14)	0.0299 (15)	0.0280 (14)	0.0170 (12)	0.0071 (11)	0.0072 (11)
C40	0.0296 (16)	0.0451 (19)	0.0368 (16)	0.0135 (14)	0.0137 (12)	0.0138 (13)
C41	0.0352 (17)	0.0419 (18)	0.0256 (14)	0.0057 (14)	0.0027 (12)	0.0023 (12)
C42	0.0490 (19)	0.0379 (17)	0.0203 (13)	0.0196 (14)	0.0074 (12)	0.0088 (12)
C43	0.0399 (17)	0.0237 (15)	0.0342 (15)	0.0115 (13)	0.0138 (12)	0.0055 (12)
C44	0.0299 (15)	0.0321 (16)	0.0232 (13)	0.0129 (13)	0.0006 (11)	−0.0004 (11)
C45	0.0517 (19)	0.0356 (17)	0.0189 (13)	0.0081 (14)	0.0042 (12)	0.0072 (12)
C46	0.0436 (18)	0.0348 (17)	0.0306 (15)	−0.0025 (14)	0.0094 (13)	0.0080 (13)
C47	0.0317 (15)	0.0265 (15)	0.0263 (14)	0.0110 (12)	0.0095 (11)	0.0081 (11)
C48	0.0317 (15)	0.0258 (14)	0.0204 (13)	0.0075 (12)	0.0028 (10)	0.0071 (10)
C49	0.0273 (14)	0.0262 (14)	0.0262 (13)	0.0082 (11)	0.0066 (11)	0.0092 (11)
C50	0.0329 (15)	0.0262 (14)	0.0214 (13)	0.0085 (12)	0.0095 (11)	0.0094 (10)
C51	0.0329 (15)	0.0313 (16)	0.0229 (13)	0.0052 (12)	0.0039 (11)	0.0105 (11)
C52	0.0257 (14)	0.0303 (15)	0.0301 (14)	0.0084 (12)	0.0052 (11)	0.0108 (11)
C53	0.040 (2)	0.113 (4)	0.067 (3)	0.030 (2)	−0.0079 (19)	−0.030 (3)
C54	0.053 (2)	0.057 (2)	0.0446 (19)	0.0150 (18)	0.0175 (16)	0.0084 (16)
C55	0.083 (4)	0.187 (7)	0.093 (4)	0.039 (4)	−0.027 (3)	−0.082 (4)
C56	0.046 (2)	0.0370 (18)	0.0321 (16)	0.0078 (15)	−0.0047 (14)	0.0058 (13)
C57	0.0376 (19)	0.052 (2)	0.059 (2)	0.0071 (16)	−0.0016 (15)	0.0237 (17)
C58	0.071 (3)	0.108 (4)	0.066 (3)	−0.019 (3)	0.036 (2)	−0.018 (3)

### Geometric parameters (Å, °)

**Table:**

Co—N1	2.083 (2)	C13—H13A	0.9500
Co—N3	2.099 (2)	C14—C15	1.378 (4)
Co—N7	2.108 (2)	C14—H14A	0.9500
Co—N5	2.174 (2)	C15—C16	1.391 (4)
Co—O2	2.1961 (17)	C15—H15A	0.9500
Co—O1	2.2872 (16)	C17—C18	1.501 (4)
O1—C9	1.413 (3)	C17—H17A	0.9900
O1—C8	1.429 (3)	C17—H17B	0.9900
O2—C29	1.419 (3)	C18—H18A	0.9800
O2—C28	1.434 (3)	C18—H18B	0.9800
O3—C41	1.232 (4)	C18—H18C	0.9800
O4—N9	1.230 (4)	C19—C20	1.510 (4)
O5—N9	1.217 (5)	C19—H19A	0.9900
O6—N10	1.222 (4)	C19—H19B	0.9900
O7—N10	1.237 (4)	C20—H20A	0.9800
O8—N11	1.249 (3)	C20—H20B	0.9800
O9—N11	1.230 (3)	C20—H20C	0.9800
O8'—N11	1.271 (3)	C21—C26	1.395 (3)
O9'—N11	1.223 (3)	C21—C22	1.399 (4)
O10—C47	1.236 (3)	C22—C23	1.376 (4)
O11—N12	1.225 (3)	C22—H22A	0.9500
O12—N12	1.227 (3)	C23—C24	1.402 (4)
O13—O13	0.000 (5)	C23—H23A	0.9500
O13—N13	1.234 (3)	C24—C25	1.384 (4)
O14—O14	0.000 (5)	C24—H24A	0.9500
O14—N13	1.243 (3)	C25—C26	1.389 (4)
O15—N14	1.223 (3)	C25—H25A	0.9500
O16—N14	1.226 (3)	C27—C28	1.485 (4)
O17—C53	1.206 (4)	C28—H28A	0.9900
O18—C56	1.216 (4)	C28—H28B	0.9900
N1—C7	1.326 (3)	C29—C30	1.493 (3)
N1—C1	1.396 (3)	C29—H29A	0.9900
N2—C7	1.348 (3)	C29—H29B	0.9900
N2—C6	1.395 (3)	C31—C32	1.384 (4)
N2—C17	1.484 (3)	C31—C36	1.399 (4)
N3—C10	1.323 (3)	C32—C33	1.378 (4)
N3—C16	1.391 (3)	C32—H32A	0.9500
N4—C10	1.343 (3)	C33—C34	1.404 (4)
N4—C11	1.391 (3)	C33—H33A	0.9500
N4—C19	1.468 (3)	C34—C35	1.382 (4)
N5—C27	1.325 (3)	C34—H34A	0.9500
N5—C21	1.389 (3)	C35—C36	1.392 (3)
N6—C27	1.353 (3)	C35—H35A	0.9500
N6—C26	1.387 (3)	C37—C38	1.513 (4)
N6—C37	1.472 (3)	C37—H37A	0.9900
N7—C30	1.321 (3)	C37—H37B	0.9900
N7—C36	1.404 (3)	C38—H38A	0.9800
N8—C30	1.346 (3)	C38—H38B	0.9800
N8—C31	1.391 (3)	C38—H38C	0.9800
N8—C39	1.472 (3)	C39—C40	1.507 (4)
N9—C42	1.455 (4)	C39—H39A	0.9900
N10—C44	1.444 (4)	C39—H39B	0.9900
N11—C46	1.463 (4)	C40—H40A	0.9800
N12—C48	1.456 (3)	C40—H40B	0.9800
N13—O13	1.234 (3)	C40—H40C	0.9800
N13—O14	1.243 (3)	C41—C46	1.452 (4)
N13—C50	1.432 (3)	C41—C42	1.452 (4)
N14—C52	1.454 (4)	C42—C43	1.367 (4)
N15—C53	1.324 (5)	C43—C44	1.383 (4)
N15—C54	1.405 (5)	C43—H43A	0.9500
N15—C55	1.455 (5)	C44—C45	1.375 (4)
N16—C56	1.327 (4)	C45—C46	1.366 (4)
N16—C58	1.450 (5)	C45—H45A	0.9500
N16—C57	1.455 (4)	C47—C48	1.453 (4)
C1—C2	1.400 (4)	C47—C52	1.456 (4)
C1—C6	1.400 (3)	C48—C49	1.362 (4)
C2—C3	1.372 (4)	C49—C50	1.393 (4)
C2—H2A	0.9500	C49—H49A	0.9500
C3—C4	1.407 (4)	C50—C51	1.388 (4)
C3—H3A	0.9500	C51—C52	1.368 (4)
C4—C5	1.387 (4)	C51—H51A	0.9500
C4—H4A	0.9500	C53—H53	0.9500
C5—C6	1.386 (4)	C54—H54A	0.9800
C5—H5A	0.9500	C54—H54B	0.9800
C7—C8	1.490 (3)	C54—H54C	0.9800
C8—H8A	0.9900	C55—H55A	0.9800
C8—H8B	0.9900	C55—H55B	0.9800
C9—C10	1.498 (3)	C55—H55C	0.9800
C9—H9A	0.9900	C56—H56A	0.9500
C9—H9B	0.9900	C57—H57A	0.9800
C11—C12	1.388 (4)	C57—H57B	0.9800
C11—C16	1.396 (4)	C57—H57C	0.9800
C12—C13	1.390 (4)	C58—H58A	0.9800
C12—H12A	0.9500	C58—H58B	0.9800
C13—C14	1.399 (4)	C58—H58C	0.9800
			
N1—Co—N3	140.20 (8)	N4—C19—H19B	109.3
N1—Co—N7	99.60 (8)	C20—C19—H19B	109.3
N3—Co—N7	100.49 (8)	H19A—C19—H19B	107.9
N1—Co—N5	97.14 (8)	C19—C20—H20A	109.5
N3—Co—N5	85.56 (8)	C19—C20—H20B	109.5
N7—Co—N5	144.80 (7)	H20A—C20—H20B	109.5
N1—Co—O2	103.97 (8)	C19—C20—H20C	109.5
N3—Co—O2	114.58 (7)	H20A—C20—H20C	109.5
N7—Co—O2	73.35 (7)	H20B—C20—H20C	109.5
N5—Co—O2	72.66 (7)	N5—C21—C26	109.4 (2)
N1—Co—O1	72.54 (7)	N5—C21—C22	130.8 (2)
N3—Co—O1	71.85 (7)	C26—C21—C22	119.8 (2)
N7—Co—O1	94.77 (7)	C23—C22—C21	117.5 (3)
N5—Co—O1	119.81 (7)	C23—C22—H22A	121.3
O2—Co—O1	167.10 (7)	C21—C22—H22A	121.3
C9—O1—C8	114.91 (18)	C22—C23—C24	122.0 (3)
C9—O1—Co	120.08 (13)	C22—C23—H23A	119.0
C8—O1—Co	119.10 (14)	C24—C23—H23A	119.0
C29—O2—C28	113.99 (18)	C25—C24—C23	121.4 (3)
C29—O2—Co	120.85 (14)	C25—C24—H24A	119.3
C28—O2—Co	122.57 (15)	C23—C24—H24A	119.3
O13—O13—N13	0(10)	C24—C25—C26	116.1 (3)
O14—O14—N13	0(10)	C24—C25—H25A	121.9
C7—N1—C1	104.94 (19)	C26—C25—H25A	121.9
C7—N1—Co	120.31 (17)	N6—C26—C25	130.9 (2)
C1—N1—Co	134.50 (15)	N6—C26—C21	105.9 (2)
C7—N2—C6	106.7 (2)	C25—C26—C21	123.2 (2)
C7—N2—C17	125.9 (2)	N5—C27—N6	113.1 (2)
C6—N2—C17	127.1 (2)	N5—C27—C28	123.5 (2)
C10—N3—C16	105.6 (2)	N6—C27—C28	123.2 (2)
C10—N3—Co	120.51 (16)	O2—C28—C27	104.38 (19)
C16—N3—Co	133.02 (16)	O2—C28—H28A	110.9
C10—N4—C11	106.8 (2)	C27—C28—H28A	110.9
C10—N4—C19	125.9 (2)	O2—C28—H28B	110.9
C11—N4—C19	127.0 (2)	C27—C28—H28B	110.9
C27—N5—C21	105.0 (2)	H28A—C28—H28B	108.9
C27—N5—Co	116.83 (17)	O2—C29—C30	104.42 (19)
C21—N5—Co	137.15 (16)	O2—C29—H29A	110.9
C27—N6—C26	106.5 (2)	C30—C29—H29A	110.9
C27—N6—C37	126.9 (2)	O2—C29—H29B	110.9
C26—N6—C37	126.2 (2)	C30—C29—H29B	110.9
C30—N7—C36	105.0 (2)	H29A—C29—H29B	108.9
C30—N7—Co	117.67 (16)	N7—C30—N8	113.7 (2)
C36—N7—Co	136.96 (16)	N7—C30—C29	122.6 (2)
C30—N8—C31	106.5 (2)	N8—C30—C29	123.7 (2)
C30—N8—C39	129.1 (2)	C32—C31—N8	131.2 (2)
C31—N8—C39	124.2 (2)	C32—C31—C36	122.7 (2)
O5—N9—O4	123.9 (3)	N8—C31—C36	106.2 (2)
O5—N9—C42	118.2 (4)	C33—C32—C31	116.9 (3)
O4—N9—C42	117.9 (3)	C33—C32—H32A	121.5
O6—N10—O7	123.9 (3)	C31—C32—H32A	121.5
O6—N10—C44	118.3 (3)	C32—C33—C34	121.0 (3)
O7—N10—C44	117.9 (3)	C32—C33—H33A	119.5
O9'—N11—O9	39.6 (6)	C34—C33—H33A	119.5
O9'—N11—O8	79.5 (8)	C35—C34—C33	121.9 (2)
O9—N11—O8	113.3 (6)	C35—C34—H34A	119.0
O9'—N11—O8'	123.6 (8)	C33—C34—H34A	119.0
O9—N11—O8'	105.6 (6)	C34—C35—C36	117.4 (2)
O8—N11—O8'	84.5 (5)	C34—C35—H35A	121.3
O9'—N11—C46	119.3 (7)	C36—C35—H35A	121.3
O9—N11—C46	117.2 (5)	C35—C36—C31	120.1 (2)
O8—N11—C46	115.1 (4)	C35—C36—N7	131.4 (2)
O8'—N11—C46	116.5 (4)	C31—C36—N7	108.6 (2)
O11—N12—O12	123.9 (2)	N6—C37—C38	111.9 (2)
O11—N12—C48	118.1 (2)	N6—C37—H37A	109.2
O12—N12—C48	118.0 (2)	C38—C37—H37A	109.2
O13—N13—O13	0.0 (2)	N6—C37—H37B	109.2
O13—N13—O14	122.8 (2)	C38—C37—H37B	109.2
O13—N13—O14	122.8 (2)	H37A—C37—H37B	107.9
O13—N13—O14	122.8 (2)	C37—C38—H38A	109.5
O13—N13—O14	122.8 (2)	C37—C38—H38B	109.5
O14—N13—O14	0.00 (19)	H38A—C38—H38B	109.5
O13—N13—C50	118.6 (2)	C37—C38—H38C	109.5
O13—N13—C50	118.6 (2)	H38A—C38—H38C	109.5
O14—N13—C50	118.6 (2)	H38B—C38—H38C	109.5
O14—N13—C50	118.6 (2)	N8—C39—C40	111.6 (2)
O15—N14—O16	123.0 (3)	N8—C39—H39A	109.3
O15—N14—C52	118.2 (2)	C40—C39—H39A	109.3
O16—N14—C52	118.7 (2)	N8—C39—H39B	109.3
C53—N15—C54	120.6 (3)	C40—C39—H39B	109.3
C53—N15—C55	124.2 (4)	H39A—C39—H39B	108.0
C54—N15—C55	115.0 (3)	C39—C40—H40A	109.5
C56—N16—C58	122.2 (3)	C39—C40—H40B	109.5
C56—N16—C57	120.8 (3)	H40A—C40—H40B	109.5
C58—N16—C57	116.8 (3)	C39—C40—H40C	109.5
N1—C1—C2	130.4 (2)	H40A—C40—H40C	109.5
N1—C1—C6	109.3 (2)	H40B—C40—H40C	109.5
C2—C1—C6	120.3 (2)	O3—C41—C46	124.5 (3)
C3—C2—C1	117.4 (2)	O3—C41—C42	125.1 (3)
C3—C2—H2A	121.3	C46—C41—C42	110.4 (2)
C1—C2—H2A	121.3	C43—C42—C41	124.9 (3)
C2—C3—C4	122.0 (2)	C43—C42—N9	116.5 (3)
C2—C3—H3A	119.0	C41—C42—N9	118.5 (3)
C4—C3—H3A	119.0	C42—C43—C44	119.5 (3)
C5—C4—C3	121.2 (3)	C42—C43—H43A	120.2
C5—C4—H4A	119.4	C44—C43—H43A	120.2
C3—C4—H4A	119.4	C45—C44—C43	120.3 (3)
C6—C5—C4	116.6 (2)	C45—C44—N10	119.5 (3)
C6—C5—H5A	121.7	C43—C44—N10	120.1 (3)
C4—C5—H5A	121.7	C46—C45—C44	119.8 (3)
C5—C6—N2	131.9 (2)	C46—C45—H45A	120.1
C5—C6—C1	122.6 (2)	C44—C45—H45A	120.1
N2—C6—C1	105.6 (2)	C45—C46—C41	125.0 (3)
N1—C7—N2	113.5 (2)	C45—C46—N11	113.4 (3)
N1—C7—C8	122.8 (2)	C41—C46—N11	121.6 (3)
N2—C7—C8	123.7 (2)	O10—C47—C48	124.0 (2)
O1—C8—C7	104.67 (18)	O10—C47—C52	125.0 (3)
O1—C8—H8A	110.8	C48—C47—C52	110.9 (2)
C7—C8—H8A	110.8	C49—C48—C47	125.3 (2)
O1—C8—H8B	110.8	C49—C48—N12	117.3 (2)
C7—C8—H8B	110.8	C47—C48—N12	117.3 (2)
H8A—C8—H8B	108.9	C48—C49—C50	118.7 (3)
O1—C9—C10	104.92 (19)	C48—C49—H49A	120.6
O1—C9—H9A	110.8	C50—C49—H49A	120.6
C10—C9—H9A	110.8	C51—C50—C49	120.9 (2)
O1—C9—H9B	110.8	C51—C50—N13	120.1 (2)
C10—C9—H9B	110.8	C49—C50—N13	119.0 (2)
H9A—C9—H9B	108.8	C52—C51—C50	119.4 (2)
N3—C10—N4	112.9 (2)	C52—C51—H51A	120.3
N3—C10—C9	122.2 (2)	C50—C51—H51A	120.3
N4—C10—C9	124.8 (2)	C51—C52—N14	117.7 (2)
C12—C11—N4	131.8 (2)	C51—C52—C47	124.4 (3)
C12—C11—C16	122.3 (2)	N14—C52—C47	117.8 (2)
N4—C11—C16	105.9 (2)	O17—C53—N15	127.5 (4)
C11—C12—C13	116.0 (3)	O17—C53—H53	116.3
C11—C12—H12A	122.0	N15—C53—H53	116.3
C13—C12—H12A	122.0	N15—C54—H54A	109.5
C12—C13—C14	122.2 (2)	N15—C54—H54B	109.5
C12—C13—H13A	118.9	H54A—C54—H54B	109.5
C14—C13—H13A	118.9	N15—C54—H54C	109.5
C15—C14—C13	121.2 (2)	H54A—C54—H54C	109.5
C15—C14—H14A	119.4	H54B—C54—H54C	109.5
C13—C14—H14A	119.4	N15—C55—H55A	109.5
C14—C15—C16	117.4 (3)	N15—C55—H55B	109.5
C14—C15—H15A	121.3	H55A—C55—H55B	109.5
C16—C15—H15A	121.3	N15—C55—H55C	109.5
C15—C16—N3	130.4 (2)	H55A—C55—H55C	109.5
C15—C16—C11	120.9 (2)	H55B—C55—H55C	109.5
N3—C16—C11	108.7 (2)	O18—C56—N16	126.1 (3)
N2—C17—C18	112.7 (2)	O18—C56—H56A	117.0
N2—C17—H17A	109.0	N16—C56—H56A	117.0
C18—C17—H17A	109.0	N16—C57—H57A	109.5
N2—C17—H17B	109.0	N16—C57—H57B	109.5
C18—C17—H17B	109.0	H57A—C57—H57B	109.5
H17A—C17—H17B	107.8	N16—C57—H57C	109.5
C17—C18—H18A	109.5	H57A—C57—H57C	109.5
C17—C18—H18B	109.5	H57B—C57—H57C	109.5
H18A—C18—H18B	109.5	N16—C58—H58A	109.5
C17—C18—H18C	109.5	N16—C58—H58B	109.5
H18A—C18—H18C	109.5	H58A—C58—H58B	109.5
H18B—C18—H18C	109.5	N16—C58—H58C	109.5
N4—C19—C20	111.7 (2)	H58A—C58—H58C	109.5
N4—C19—H19A	109.3	H58B—C58—H58C	109.5
C20—C19—H19A	109.3		
			
N1—Co—O1—C9	−155.93 (19)	Co—N5—C21—C26	168.72 (18)
N3—Co—O1—C9	6.00 (17)	C27—N5—C21—C22	−178.8 (3)
N7—Co—O1—C9	105.49 (18)	Co—N5—C21—C22	−11.2 (4)
N5—Co—O1—C9	−67.64 (19)	N5—C21—C22—C23	−179.5 (3)
O2—Co—O1—C9	128.0 (3)	C26—C21—C22—C23	0.6 (4)
N1—Co—O1—C8	−4.36 (17)	C21—C22—C23—C24	−0.6 (4)
N3—Co—O1—C8	157.56 (18)	C22—C23—C24—C25	0.0 (5)
N7—Co—O1—C8	−102.94 (17)	C23—C24—C25—C26	0.4 (4)
N5—Co—O1—C8	83.92 (18)	C27—N6—C26—C25	179.1 (3)
O2—Co—O1—C8	−80.5 (4)	C37—N6—C26—C25	5.1 (5)
N1—Co—O2—C29	−106.16 (18)	C27—N6—C26—C21	−0.6 (3)
N3—Co—O2—C29	84.01 (19)	C37—N6—C26—C21	−174.5 (2)
N7—Co—O2—C29	−10.14 (17)	C24—C25—C26—N6	179.9 (3)
N5—Co—O2—C29	160.60 (19)	C24—C25—C26—C21	−0.4 (4)
O1—Co—O2—C29	−33.6 (4)	N5—C21—C26—N6	−0.3 (3)
N1—Co—O2—C28	93.21 (19)	C22—C21—C26—N6	179.6 (2)
N3—Co—O2—C28	−76.6 (2)	N5—C21—C26—C25	180.0 (2)
N7—Co—O2—C28	−170.8 (2)	C22—C21—C26—C25	−0.1 (4)
N5—Co—O2—C28	−0.04 (18)	C21—N5—C27—N6	−1.6 (3)
O1—Co—O2—C28	165.8 (3)	Co—N5—C27—N6	−172.15 (16)
N3—Co—N1—C7	−28.3 (2)	C21—N5—C27—C28	174.0 (2)
N7—Co—N1—C7	91.11 (19)	Co—N5—C27—C28	3.5 (3)
N5—Co—N1—C7	−119.97 (18)	C26—N6—C27—N5	1.4 (3)
O2—Co—N1—C7	166.19 (18)	C37—N6—C27—N5	175.3 (2)
O1—Co—N1—C7	−0.91 (17)	C26—N6—C27—C28	−174.2 (2)
N3—Co—N1—C1	158.5 (2)	C37—N6—C27—C28	−0.3 (4)
N7—Co—N1—C1	−82.1 (2)	C29—O2—C28—C27	−160.4 (2)
N5—Co—N1—C1	66.8 (2)	Co—O2—C28—C27	1.5 (3)
O2—Co—N1—C1	−7.0 (2)	N5—C27—C28—O2	−3.2 (3)
O1—Co—N1—C1	−174.1 (2)	N6—C27—C28—O2	172.0 (2)
N1—Co—N3—C10	21.8 (3)	C28—O2—C29—C30	171.8 (2)
N7—Co—N3—C10	−97.34 (19)	Co—O2—C29—C30	9.6 (3)
N5—Co—N3—C10	117.65 (19)	C36—N7—C30—N8	−0.1 (3)
O2—Co—N3—C10	−173.70 (17)	Co—N7—C30—N8	174.38 (16)
O1—Co—N3—C10	−5.72 (18)	C36—N7—C30—C29	179.7 (2)
N1—Co—N3—C16	−146.1 (2)	Co—N7—C30—C29	−5.9 (3)
N7—Co—N3—C16	94.8 (2)	C31—N8—C30—N7	−0.6 (3)
N5—Co—N3—C16	−50.2 (2)	C39—N8—C30—N7	−175.7 (2)
O2—Co—N3—C16	18.4 (2)	C31—N8—C30—C29	179.6 (2)
O1—Co—N3—C16	−173.6 (2)	C39—N8—C30—C29	4.5 (4)
N1—Co—N5—C27	−104.17 (18)	O2—C29—C30—N7	−2.4 (3)
N3—Co—N5—C27	115.76 (18)	O2—C29—C30—N8	177.3 (2)
N7—Co—N5—C27	13.8 (3)	C30—N8—C31—C32	−179.7 (3)
O2—Co—N5—C27	−1.72 (17)	C39—N8—C31—C32	−4.2 (4)
O1—Co—N5—C27	−178.11 (16)	C30—N8—C31—C36	1.1 (3)
N1—Co—N5—C21	89.3 (2)	C39—N8—C31—C36	176.5 (2)
N3—Co—N5—C21	−50.8 (2)	N8—C31—C32—C33	−178.2 (3)
N7—Co—N5—C21	−152.7 (2)	C36—C31—C32—C33	0.9 (4)
O2—Co—N5—C21	−168.3 (2)	C31—C32—C33—C34	0.1 (4)
O1—Co—N5—C21	15.3 (3)	C32—C33—C34—C35	−0.1 (4)
N1—Co—N7—C30	109.79 (18)	C33—C34—C35—C36	−0.8 (4)
N3—Co—N7—C30	−104.75 (18)	C34—C35—C36—C31	1.7 (4)
N5—Co—N7—C30	−7.5 (3)	C34—C35—C36—N7	−179.9 (3)
O2—Co—N7—C30	7.96 (17)	C32—C31—C36—C35	−1.9 (4)
O1—Co—N7—C30	−177.14 (17)	N8—C31—C36—C35	177.5 (2)
N1—Co—N7—C36	−78.0 (2)	C32—C31—C36—N7	179.5 (2)
N3—Co—N7—C36	67.5 (2)	N8—C31—C36—N7	−1.2 (3)
N5—Co—N7—C36	164.7 (2)	C30—N7—C36—C35	−177.6 (3)
O2—Co—N7—C36	−179.8 (2)	Co—N7—C36—C35	9.5 (4)
O1—Co—N7—C36	−4.9 (2)	C30—N7—C36—C31	0.8 (3)
O13—O13—N13—O14	0.00 (19)	Co—N7—C36—C31	−172.02 (18)
O13—O13—N13—O14	0.00 (19)	C27—N6—C37—C38	−95.6 (3)
O13—O13—N13—C50	0.00 (14)	C26—N6—C37—C38	77.1 (3)
O14—O14—N13—O13	0.00 (10)	C30—N8—C39—C40	101.1 (3)
O14—O14—N13—O13	0.00 (10)	C31—N8—C39—C40	−73.2 (3)
O14—O14—N13—C50	0.00 (15)	O3—C41—C42—C43	175.2 (3)
C7—N1—C1—C2	178.0 (3)	C46—C41—C42—C43	−1.8 (4)
Co—N1—C1—C2	−8.1 (4)	O3—C41—C42—N9	−1.4 (5)
C7—N1—C1—C6	−0.5 (3)	C46—C41—C42—N9	−178.4 (3)
Co—N1—C1—C6	173.48 (17)	O5—N9—C42—C43	142.6 (3)
N1—C1—C2—C3	179.9 (2)	O4—N9—C42—C43	−37.0 (4)
C6—C1—C2—C3	−1.9 (4)	O5—N9—C42—C41	−40.5 (4)
C1—C2—C3—C4	0.7 (4)	O4—N9—C42—C41	139.9 (3)
C2—C3—C4—C5	0.6 (4)	C41—C42—C43—C44	3.3 (4)
C3—C4—C5—C6	−0.7 (4)	N9—C42—C43—C44	180.0 (3)
C4—C5—C6—N2	179.2 (3)	C42—C43—C44—C45	−2.9 (4)
C4—C5—C6—C1	−0.5 (4)	C42—C43—C44—N10	−178.8 (2)
C7—N2—C6—C5	179.6 (3)	O6—N10—C44—C45	179.9 (3)
C17—N2—C6—C5	−6.3 (4)	O7—N10—C44—C45	−0.9 (4)
C7—N2—C6—C1	−0.6 (3)	O6—N10—C44—C43	−4.2 (4)
C17—N2—C6—C1	173.5 (2)	O7—N10—C44—C43	175.0 (2)
N1—C1—C6—C5	−179.5 (2)	C43—C44—C45—C46	1.1 (4)
C2—C1—C6—C5	1.9 (4)	N10—C44—C45—C46	177.0 (3)
N1—C1—C6—N2	0.7 (3)	C44—C45—C46—C41	0.4 (5)
C2—C1—C6—N2	−177.9 (2)	C44—C45—C46—N11	−176.1 (3)
C1—N1—C7—N2	0.0 (3)	O3—C41—C46—C45	−177.1 (3)
Co—N1—C7—N2	−174.95 (16)	C42—C41—C46—C45	−0.1 (4)
C1—N1—C7—C8	−178.9 (2)	O3—C41—C46—N11	−0.9 (5)
Co—N1—C7—C8	6.1 (3)	C42—C41—C46—N11	176.1 (3)
C6—N2—C7—N1	0.4 (3)	O9'—N11—C46—C45	−150.0 (9)
C17—N2—C7—N1	−173.8 (2)	O9—N11—C46—C45	164.8 (6)
C6—N2—C7—C8	179.3 (2)	O8—N11—C46—C45	−58.3 (6)
C17—N2—C7—C8	5.1 (4)	O8'—N11—C46—C45	38.4 (5)
C9—O1—C8—C7	160.6 (2)	O9'—N11—C46—C41	33.4 (10)
Co—O1—C8—C7	7.6 (2)	O9—N11—C46—C41	−11.7 (7)
N1—C7—C8—O1	−8.8 (3)	O8—N11—C46—C41	125.2 (5)
N2—C7—C8—O1	172.4 (2)	O8'—N11—C46—C41	−138.2 (5)
C8—O1—C9—C10	−157.7 (2)	O10—C47—C48—C49	169.2 (3)
Co—O1—C9—C10	−5.0 (2)	C52—C47—C48—C49	−6.8 (4)
C16—N3—C10—N4	−1.1 (3)	O10—C47—C48—N12	−7.7 (4)
Co—N3—C10—N4	−171.93 (16)	C52—C47—C48—N12	176.3 (2)
C16—N3—C10—C9	176.2 (2)	O11—N12—C48—C49	140.6 (3)
Co—N3—C10—C9	5.4 (3)	O12—N12—C48—C49	−39.2 (4)
C11—N4—C10—N3	1.3 (3)	O11—N12—C48—C47	−42.2 (4)
C19—N4—C10—N3	175.7 (2)	O12—N12—C48—C47	138.0 (3)
C11—N4—C10—C9	−176.0 (2)	C47—C48—C49—C50	4.0 (4)
C19—N4—C10—C9	−1.5 (4)	N12—C48—C49—C50	−179.1 (2)
O1—C9—C10—N3	0.0 (3)	C48—C49—C50—C51	0.0 (4)
O1—C9—C10—N4	177.0 (2)	C48—C49—C50—N13	179.5 (2)
C10—N4—C11—C12	178.7 (3)	O13—N13—C50—C51	−178.5 (3)
C19—N4—C11—C12	4.3 (4)	O13—N13—C50—C51	−178.5 (3)
C10—N4—C11—C16	−0.9 (3)	O14—N13—C50—C51	0.6 (4)
C19—N4—C11—C16	−175.3 (2)	O14—N13—C50—C51	0.6 (4)
N4—C11—C12—C13	−179.5 (3)	O13—N13—C50—C49	1.9 (4)
C16—C11—C12—C13	0.1 (4)	O13—N13—C50—C49	1.9 (4)
C11—C12—C13—C14	−0.2 (4)	O14—N13—C50—C49	−178.9 (2)
C12—C13—C14—C15	0.1 (5)	O14—N13—C50—C49	−178.9 (2)
C13—C14—C15—C16	0.3 (4)	C49—C50—C51—C52	−0.3 (4)
C14—C15—C16—N3	179.1 (3)	N13—C50—C51—C52	−179.8 (3)
C14—C15—C16—C11	−0.5 (4)	C50—C51—C52—N14	−179.9 (3)
C10—N3—C16—C15	−179.2 (3)	C50—C51—C52—C47	−3.3 (4)
Co—N3—C16—C15	−10.0 (4)	O15—N14—C52—C51	33.4 (4)
C10—N3—C16—C11	0.5 (3)	O16—N14—C52—C51	−145.2 (3)
Co—N3—C16—C11	169.68 (17)	O15—N14—C52—C47	−143.4 (3)
C12—C11—C16—C15	0.3 (4)	O16—N14—C52—C47	38.0 (4)
N4—C11—C16—C15	180.0 (2)	O10—C47—C52—C51	−169.6 (3)
C12—C11—C16—N3	−179.4 (2)	C48—C47—C52—C51	6.4 (4)
N4—C11—C16—N3	0.2 (3)	O10—C47—C52—N14	7.0 (4)
C7—N2—C17—C18	−110.1 (3)	C48—C47—C52—N14	−177.0 (2)
C6—N2—C17—C18	76.9 (3)	C54—N15—C53—O17	−1.8 (9)
C10—N4—C19—C20	−86.1 (3)	C55—N15—C53—O17	−176.0 (7)
C11—N4—C19—C20	87.2 (3)	C58—N16—C56—O18	−176.6 (4)
C27—N5—C21—C26	1.1 (3)	C57—N16—C56—O18	−1.4 (5)

### Hydrogen-bond geometry (Å, °)

**Table:**

*D*—H···*A*	*D*—H	H···*A*	*D*···*A*	*D*—H···*A*
C18—H18B···O12^i^	0.98	2.57	3.342 (4)	135
C15—H15A···O13	0.95	2.56	3.191 (3)	124
C18—H18B···O12^i^	0.98	2.57	3.342 (4)	135
C28—H28A···O17^ii^	0.99	2.38	3.365 (4)	173
C28—H28B···O10^iii^	0.99	2.29	2.994 (3)	128
C29—H29A···O14	0.99	2.39	3.331 (3)	158
C35—H35A···O7^i^	0.95	2.44	3.158 (3)	133
C53—H53···O9'^iv^	0.95	2.39	3.242 (7)	148
C56—H56A···O3^v^	0.95	2.46	3.335 (4)	154

Symmetry codes: (i) *x*, *y*, *z*+1;  (ii) *x*, *y*−1, *z*; (iii) −*x*+1, −*y*, −*z*; (iv) *x*−1, *y*, *z*; (v) −*x*+1, −*y*+1, −*z*+1.
